# Endothelial-specific Gata3 expression is required for hematopoietic stem cell generation

**DOI:** 10.1016/j.stemcr.2022.06.008

**Published:** 2022-07-28

**Authors:** Nada Zaidan, Leslie Nitsche, Evangelia Diamanti, Rebecca Hannah, Antonella Fidanza, Nicola K. Wilson, Lesley M. Forrester, Berthold Göttgens, Katrin Ottersbach

**Affiliations:** 1 Centre for Regenerative Medicine, Institute for Regeneration and Repair, University of Edinburgh, Edinburgh EH16 4UU, UK; 2 Department of Haematology, Wellcome Trust-Medical Research Council Cambridge Stem Cell Institute, University of Cambridge, Cambridge CB2 0AW, UK

**Keywords:** hematopoietic stem cell, Gata3, hemogenic endothelial cells, endothelial-to-hematopoietic transition, aorta-gonads-mesonephros, cell cycle, *Cdkn1c*, *p57Kip2*

## Abstract

To generate sufficient numbers of transplantable hematopoietic stem cells (HSCs) *in vitro*, a detailed understanding of how this process takes place *in vivo* is essential. The endothelial-to-hematopoietic transition (EHT), which culminates in the production of the first HSCs, is a highly complex process during which key regulators are switched on and off at precise moments, and that is embedded into a myriad of microenvironmental signals from surrounding cells and tissues. We have previously demonstrated an HSC-supportive function for GATA3 within the sympathetic nervous system and the sub-aortic mesenchyme, but show here that it also plays a cell-intrinsic role during the EHT. It is expressed in hemogenic endothelial cells and early HSC precursors, where its expression correlates with a more quiescent state. Importantly, endothelial-specific deletion of *Gata3* shows that it is functionally required for these cells to mature into HSCs, placing GATA3 at the core of the EHT regulatory network.

## Highlights

• *Gata3* marks hemogenic endothelial cells and early HSC precursors
• *Gata3* expression correlates with early EHT drivers
• *Gata3* is associated with a quiescent cell state
• GATA3 is essential for the endothelial-to-hematopoietic transition

## Teaser

Ottersbach and colleagues demonstrate that *Gata3* marks hemogenic endothelial cells and early hematopoietic stem cell precursors where its expression correlates with other early drivers of the endothelial-to-hematopoietic transition and a quiescent cell cycle state. Importantly, they show that GATA3 facilitates the transition of hemogenic endothelial cells into fully functional hematopoietic stem cells.

## Introduction

The generation of the first transplantable hematopoietic stem cells (HSCs) initiates in the dorsal aorta of the aorta-gonad-mesonephros (AGM) region at embryonic day 10.5 (E10.5) in mouse embryos (de Bruijn et al., 2000; Medvinsky and Dzierzak, 1996). It involves activating a hematopoietic transcriptional program in a subset of endothelial cells (ECs), as a result of a number of internal and external signals, which drives these cells to adopting a hematopoietic fate (Ottersbach, 2019). These so-called hemogenic endothelial cells (HECs) undergo major morphological changes, resulting in the appearance of intra-aortic cell clusters in which HECs further mature from pro-HSCs to type I and type II pre-HSCs, before becoming fully mature HSCs (Rybtsov et al., 2014). While it is known that the two transcription factors RUNX1 and GATA2 are essential for the endothelial-to-hematopoietic transition (EHT) (Chen et al., 2009; de Pater et al., 2013), it is clear that they do not act alone. For example, SOX17 is required upstream for arterial fate and HEC specification (Clarke et al., 2013; Corada et al., 2013), while GFI1 and GFI1B are downstream targets of RUNX1, necessary for EHT completion (Thambyrajah et al., 2016). How all of these transcription factors interact and whether they form multi-component complexes, however, remains unknown.

In this study, we reveal the transcription factor GATA3 as another important regulator of the EHT. Its expression is upregulated in HECs and early HSC precursors, thereby enriching for hemogenic potential, but is downregulated before fully mature HSCs are formed. Through RNA sequencing (RNA-seq) analysis, we show co-expression of *Gata3* with many important EHT regulators and have been able to link *Gata3* expression with a more quiescent cell state. Importantly, endothelial-specific deletion of *Gata3* significantly reduces hematopoietic stem and progenitor cell (HSPC) formation, which, together with its hematopoiesis-supportive role in the co-developing sympathetic nervous system (SNS) (Fitch et al., 2012) and the sub-aortic mesenchyme (Fitch et al., 2020), gives GATA3 a multi-faceted, central role in HSC generation.

## Results

### GATA3 is expressed in a subset of endothelial and hematopoietic cells

**Figure 1:**
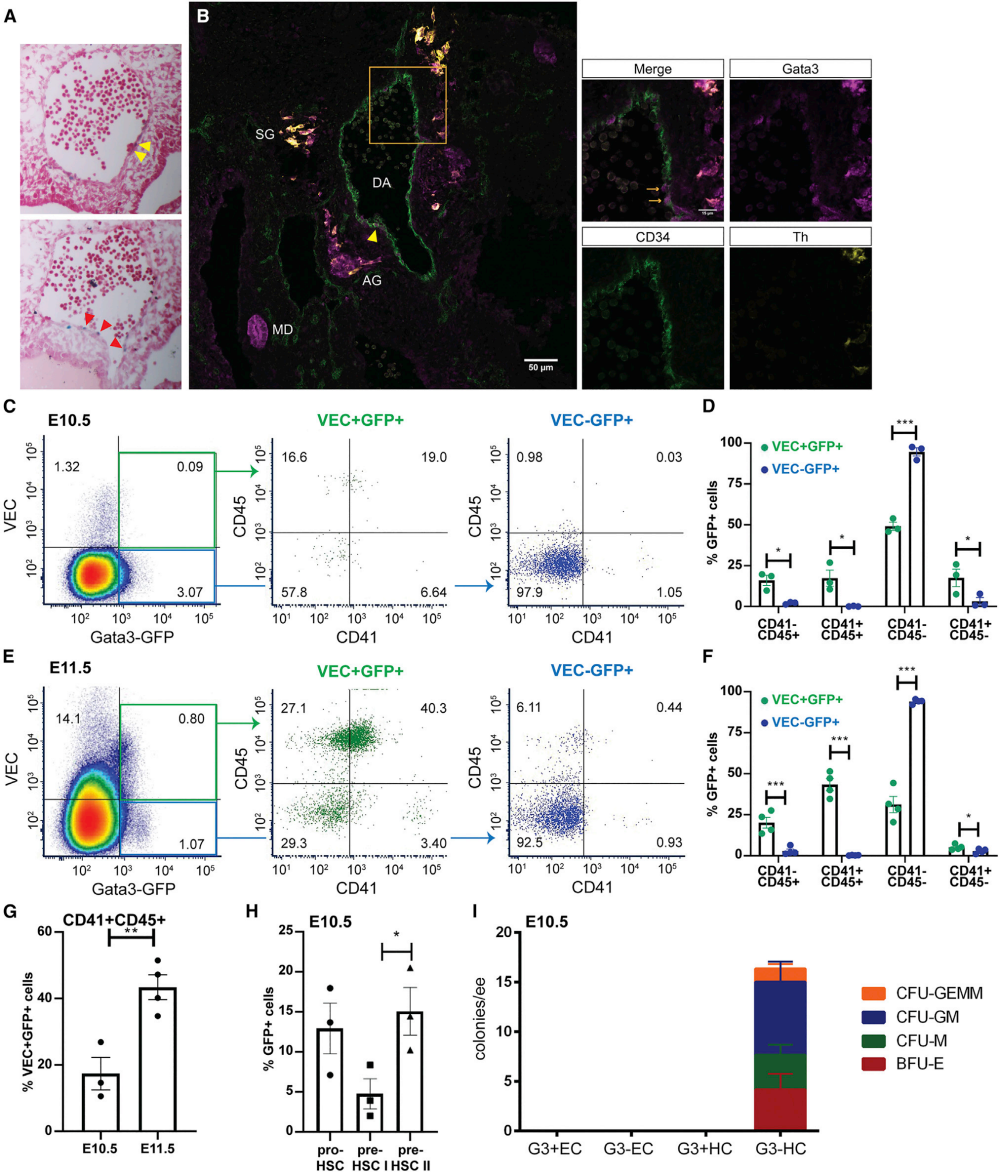
GATA3 is expressed in a subset of endothelial and hematopoietic cells (A) Cryosections of E10.5 *Gata3*^*lz/+*^ embryos with *Gata3*-LacZ expression in blue and counterstained with Neutral Red. Yellow arrowheads point to *Gata3*^+^ cells near intra-aortic clusters, red arrowheads highlight *Gata3*^+^ ECs. (B–F) Section from a *Gata3*-GFP^+^ E11.5 embryo stained with CD34 (green), *Gata3*-GFP (magenta) and TH (yellow). Orange box indicates area shown at higher magnification on the right. The yellow arrowhead points to a *Gata3*^+^ cell at the base of an intra-aortic cluster. The orange arrows in the higher magnification image highlight *Gata3*^+^ ECs. AG, adrenal anlage, DA, dorsal aorta, SG, sympathetic ganglia, MD, mesonephric duct. Flow cytometry analysis of *Gata3*-GFP^+^ E10.5 AGMs, stained for GFP, VEC, CD41, and CD45. Representative flow plots shown in (C) and results from three biological replicates shown in (D). Flow cytometry analysis of *Gata3*-GFP^+^ E11.5 AGMs, stained for GFP, VEC, CD41, and CD45. Representative flow plots shown in (E) and results from four biological replicates shown in (F). One biological replicate = 1 embryo. (G) Increase of VEC^+^GFP^+^CD41^+^CD45^+^ cells from E10.5 to E11.5. (H) Percentage of pro-HSCs (VEC^+^CD41^+^CD43^−^CD45^−^), pre-HSC type I (VEC^+^CD41^+^CD43^+^CD45^−^) and pre-HSC type II (VEC^+^CD41^+^CD43^+^CD45^+^) within the *Gata3*-GFP^+^ fraction of E10.5 AGMs. n = 3; three embryos used. An unpaired t test was performed; ^∗^p < 0.05; ^∗∗^p < 0.01; ^∗∗∗^p < 0.001. (I) CFU-C assay of freshly sorted and directly plated G3-EC: CD41/45^−^GFP^−^VEC^+^, G3+EC: CD41/45^−^GFP^+^VEC^+^, G3-HC: CD41/45^+^GFP^−^, and G3+HC: CD41/45^+^GFP^+^; three technical replicates of one biological replicate. See also Figure S1.

We had previously detected GATA3 expression in a subset of ECs (Fitch et al., 2012) (Figure 1A, red arrowheads), often in the vicinity of intra-aortic cell clusters (Figure 1A, yellow arrowheads), which suggested that GATA3 may be expressed in HECs. We carried out a more careful analysis using a *Gata3*-GFP reporter mouse line (Grote et al., 2006). Immunohistochemical co-staining for GATA3 with an anti-GATA3 and an anti-GFP antibody confirmed this construct to be a faithful reporter (Figures S1A–S1F), that recapitulated the expression of *Gata3* in individual ECs (Figure 1B, arrows) and at the base of intra-aortic clusters (Figure 1B, yellow arrowhead), but not within clusters. Flow cytometry analysis confirmed that a fraction (6.4%) of ECs (VE-CADHERIN [VEC]+) express *Gata3*-GFP at E10.5 when HEC frequency is at its highest (Figure 1C). Interestingly, more than 40% of these *Gata3*-GFP^+^ ECs also express the hematopoietic markers CD45 (late hematopoietic marker) and/or CD41 (early marker), with 17% expressing both (Figure 1D). The percentage of CD45^+^ *Gata3*-GFP^+^ cells was much lower within the VEC^−^ fraction, suggesting that the majority are VEC^+^ HSPCs or HSC precursors. This percentage increases substantially at E11.5 (Figures 1E–1G). To further investigate whether the *Gata3*-GFP^+^ fraction contains phenotypic HSC precursors, we used the nomenclature developed by the Medvinsky lab (Rybtsov et al., 2014), with pro-HSCs defined as VEC^+^CD41^+^CD43^−^CD45^−^, pre-HSC type I as VEC^+^CD41^+^CD43^+^CD45^−^and pre-HSC type II as VEC^+^CD41^+^CD43^+^CD45^+^ (Figure S1G). All three populations were present in the *Gata3*-GFP^+^ fraction (Figure 1H).

To determine if the *Gata3*-GFP^+^ hematopoietic population at E10.5 contains HSPCs, we sorted them (Figure S1H) and plated them directly in colony-forming (CFU-C) assays alongside *Gata3*-GFP^-^ hematopoietic cells (HCs) and *Gata3*-GFP^+/−^ ECs. As expected, ECs do not produce hematopoietic colonies when plated directly in methylcellulose (Figure 1I). Interestingly, only the *Gata3*-GFP^−^ HCs gave rise to colonies, but not the GFP^+^ fraction, suggesting that the latter consists either of mature hematopoietic cells or very early precursors that require further maturation toward the hematopoietic fate.

### *Gata3* expression enriches for hemogenic endothelial activity

**Figure 2:**
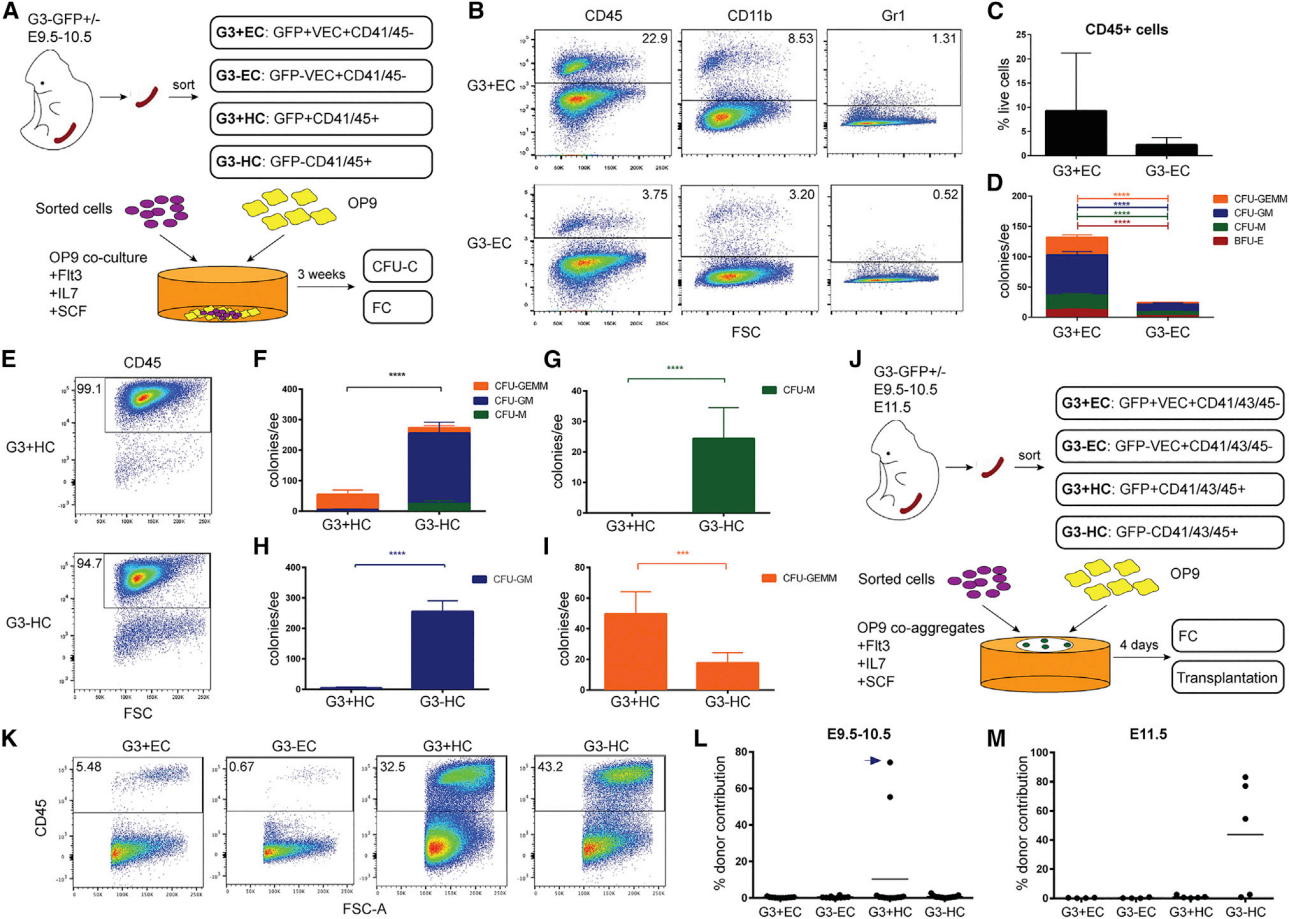
*Gata3* enriches for HECs and marks early HSC precursors (A) Outline of co-culture experiments. (B–D) Flow cytometry analysis of hematopoietic output from *Gata3*-GFP^+/−^ ECs. Total CD45^+^ cells (flow cytometry) (C) and hematopoietic progenitors (CFU-C) (D) produced by *Gata3*-GFP^+/−^ ECs; n = 4, with pooled and sorted cells from 8 to 12 embryos used per experiment. (E–I) Flow cytometry analysis of CD45^+^ cells produced from *Gata3*-GFP^+/−^ HCs from three independent experiments (n = 3). Total progenitors (F), CFU-M (G), CFU-GM (H), and CFU-GEMM (I) produced by *Gata3*-GFP^+/−^ HCs; n = 3, with pooled and sorted cells from 8 to 12 embryos used per experiment. ^∗∗∗^p < 0.001; ^∗∗∗∗^p < 0.0001; Mann-Whitney test. (J) Outline of co-aggregate experiments. (K–M) Total CD45^+^ cell output (flow cytometry) from E9.5–10.5 *Gata3*-GFP^+/−^ ECs and HCs. Percent donor contribution after 4 months in recipients of co-aggregates from E9.5–10.5 (L) and E11.5 (M) *Gata3*-GFP^+/−^ ECs and HCs. Arrow highlights data point from analysis after 1 month as this recipient died unexpectedly before the 4-month analysis. n = 7 for E9.5–10.5. Cells were sorted from 7 to 13 embryos into the four populations, which were co-aggregated as one embryo equivalent (ee), with one co-aggregate transplanted per recipient (28 recipients in total; seven per cell population); n = 4–5 for E11.5. Cells were sorted from six to 10 embryos into the indicated populations, which were co-aggregated as one ee (four co-aggregates per EC population; five co-aggregates per HC population), with one co-aggregate transplanted per recipient (18 recipients in total). See also Figures S1 and S2.

Inherent hemogenic potential can be revealed via a co-culture step on OP9 stromal cells (Swiers et al., 2013). *Gata3*-GFP^+/−^ ECs were sorted (Figure S1H) and cultured with OP9 cells. Cells were then assessed for hematopoietic marker expression and progenitor potential (Figure 2A). Both EC populations were able to give rise to HCs (Figure 2B), with a trend toward higher production of CD45^+^ cells from *Gata3*-GFP^+^ ECs (Figure 2C). In CFU-C assays, however, *Gata3*-GFP^+^ ECs had a noticeably higher colony output (Figure 2D), which was highly significant for all colony types.

### *Gata3* marks HSC precursors

We also tested the potential of the *Gata3*-GFP^+/−^ HC populations (Figures 2A and S1H) in co-cultures. Unsurprisingly, they gave an almost entirely CD45^+^ output (Figure 2E). Interestingly, while overall progenitor potential was higher in the *Gata3*-GFP^−^ fraction (Figure 2F), splitting this into individual progenitor types revealed that *Gata3* expression enriches specifically for the most immature CFU-GEMM progenitor (Figures 2G–2I), suggesting that *Gata3*-GFP may mark HSC precursors.

The intermediate steps involved in the maturation of HECs into transplantable HSCs have been dissected with the help of a culture system developed in the Medvinsky lab (Rybtsov et al., 2011, 2014). It involves aggregation of sorted cell populations with OP9 cells and culturing these co-aggregates in the presence of cytokines. The potential of the initial populations is then assessed by flow cytometry and transplantations (Figure 2J). Pro-HSCs emerge from E9 in the AGM region, while type I pre-HSCs are detected at E10–11. Pre-HSC type II emerge toward E11 (Rybtsov et al., 2014). *Gata3*-GFP^+/−^ ECs and HCs were sorted (Figure S2A) and cultured as OP9 co-aggregates (Figure 2J). All four populations were able to generate CD45^+^ HCs, with the sorted HC fractions producing substantially more CD45^+^ cells than the EC populations (Figure 2K). ECs are unable to transdifferentiate into transplantable HSCs in these culture conditions (Rybtsov et al., 2014), and indeed neither *Gata3*-GFP^+^ nor *Gata3*-GFP^−^ ECs produced any detectable chimerism in transplant recipients (Figures 2L and 2M). Intriguingly, repopulation activity at E9.5 to E10.5 was restricted to the *Gata3*-GFP^+^ HC fraction, which completely shifted to the *Gata3*-GFP^−^ fraction at E11.5. All of the results taken together with our previous data (Fitch et al., 2012) imply that *Gata3* expression is switched on in HECs, with *Gata3* expression continuing during the early stages of HSC maturation, but being switched off at the pre-HSC type II stage and remaining off in emerging HSCs (Figure S2B).

### *Gata3*-expressing cells show an enrichment for key regulators of EHT

**Figure 3:**
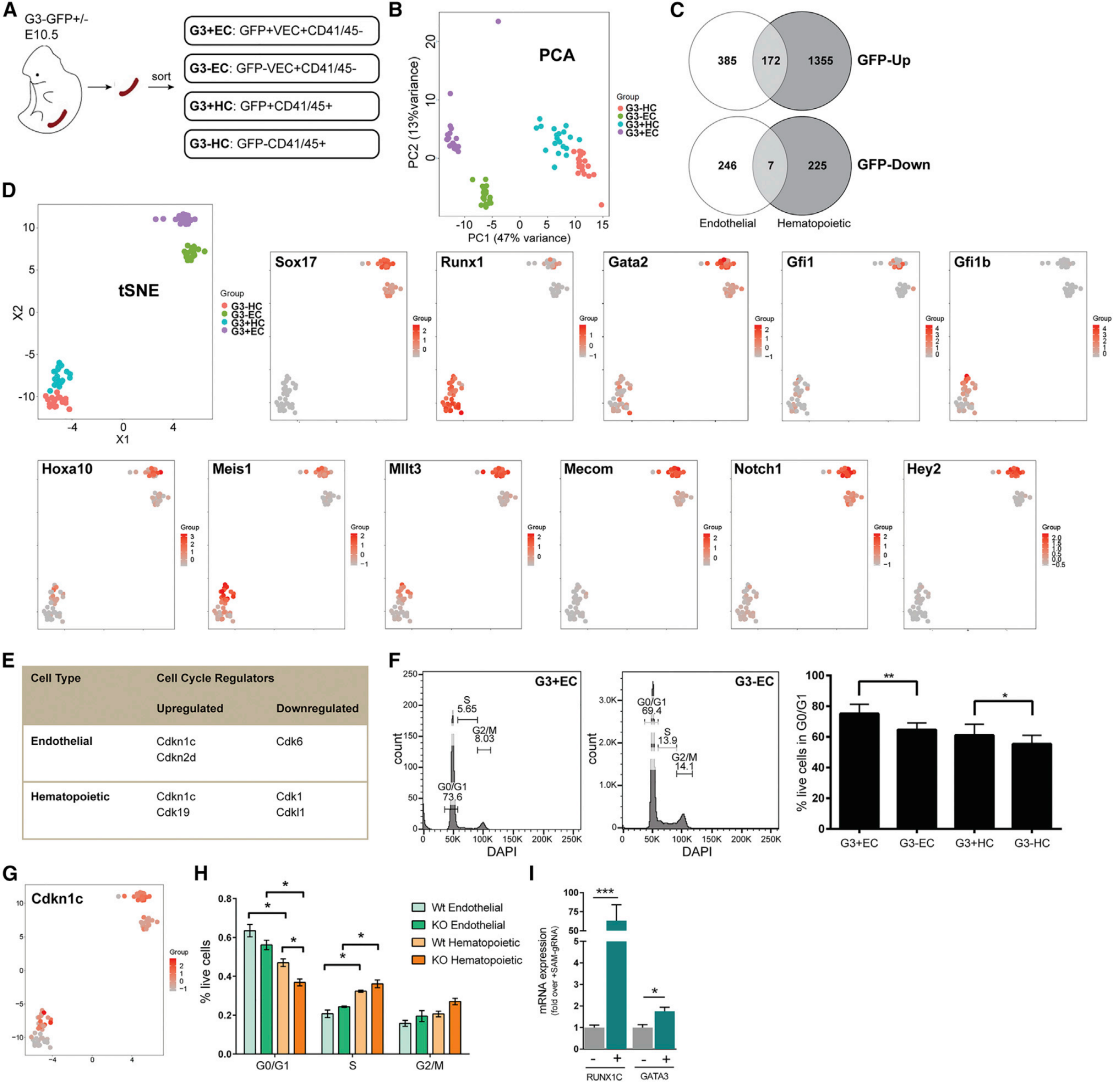
*Gata3* expression correlates with other EHT regulators and marks quiescent cells (A) Cell populations analyzed by RNA-seq. (B) Principal-component analysis of individual samples of the four populations. Cells were from three to five biological replicates, with cells from two to six GFP^+^ embryos pooled per replicate. (C) Venn diagrams of genes upregulated and downregulated in *Gata3*-GFP^+^ ECs and HCs. (D) tSNE plots of the four cell populations colored for the expression of the indicated genes. (E) Cell cycle regulators differentially expressed in *Gata3*-GFP^+^ ECs and HCs. (F) Percentage of quiescent cells in the *Gata3*-GFP^+/−^ EC and HC populations, with representative flow cytometry plots. n = 4, with cells from 2 to 10 GFP^+^ embryos pooled per independent experiment. ^∗^p < 0.05; ^∗∗^p < 0.01; paired t test. (G) tSNE plot colored for *Cdkn1c* expression. (H) Percentage of *Cdkn1c* wild-type (Wt) and knockout (KO) ECs and HCs in the different cell cycle phases. n = 3, using a total of 11 Wt (4, 4, 3) and 11 KO (4, 4, 3) embryos. ^∗^p < 0.05; paired t test. (I) Expression of *RUNX1C* and *GATA3* by qPCR in hiPSCs with (+) and without (−) *RUNX1C*-activating gRNA as part of the UniSam endogenous gene activation system. n = 3. ^∗^p < 0.05; ^∗∗∗^p < 0.001; Mann-Whitney test. See also Figures S2 and S3.

To get a better understanding of how GATA3 may be involved in the EHT, we performed RNA-seq on small pools (20 cells/pool; 20 pools/population) of sorted *Gata3*-GFP^+^ ECs and HCs and compared their transcriptome to the *Gata3*-GFP^−^ fractions (Figures 3A and S1H). Principal-component analysis showed a clear separation of ECs from HCs along component 1, with each population displaying a distinct subdivision according to *Gata3* expression along component 2 (Figure 3B). Differential expression analysis revealed a higher number of genes being upregulated in *Gata3*-GFP^+^ cells (EC: 557; HC: 1527) than were downregulated (EC: 253; HC: 232), with some overlap between the two cell types (Figure 3C; Table S1). Gene ontology analysis of the upregulated genes saw a significant enrichment of processes associated with stem cell development and differentiation and migration in both cell populations, which may be a reflection of these cells undergoing morphological changes during EHT (Figure S3). Genes that were downregulated in the *Gata3*-GFP^+^ HC fraction were largely associated with differentiated blood lineages (e.g., *Icos*, *Irf8*, *Klf4*, *Ccl3,* and *Il6ra*), which reflects the HSC precursor status of the *Gata3*-GFP^+^ cells. Interestingly, *Gata3*-GFP^+^ ECs displayed a downregulation of genes linked to the positive regulation of cell cycle and proliferation.

To understand the position of GATA3 in the network of EHT regulators, we analyzed the expression of regulators that mark key stages of HSC generation (Ottersbach, 2019) (Figure S2B). *Sox17* is upregulated in arterial ECs and required for HEC specification, but subsequently needs to be downregulated for the EHT to proceed. This is confirmed in our dataset, where it is absent in HCs, but upregulated in *Gata3*-GFP^+^ ECs (Figure 3D). *Runx1*, *Gata2,* and *Gfi1* are key transcription factors for initiating the hematopoietic transcriptional program in HEC, which is reflected in their upregulation in *Gata3*-GFP^+^ ECs, with *Gata2* showing the most widespread expression. *Runx1* is much more highly expressed in HCs (*Gata3*-GFP^+^ and ^–^HCs), while *Gata2* and *Gfi1* become downregulated, which is consistent with the literature (Ottersbach, 2019). *Gfi1b* is involved in the final stages of the EHT, with expression restricted to intra-aortic clusters. Accordingly, its expression was confined to the *Gata3*-GFP^+^ HC population.

Among the genes commonly upregulated in *Gata3*-GFP^+^ ECs and HCs (Table S1, Figure 3D) were well-known HSC regulators such as *Hoxa10*, which was shown to drive the transition of HECs into HSCs derived from human iPSCs (Sugimura et al., 2017), *Meis1*, which promotes the reprogramming of hematopoietic progenitors to HSCs (Riddell et al., 2014), and *Mllt3*, which is essential for human HSC self-renewal (Calvanese et al., 2019) and was reported to be upregulated in cells undergoing EHT (Oatley et al., 2020). *Mecom*, a transcription factor essential for fetal and adult HSC function (Kumano and Kurokawa, 2010), that was recently, together with *Hoxa10* and *Meis1*, described as a marker for E9.5 HECs (Gao et al., 2018), was upregulated in *Gata3*-GFP^+^ ECs. The Notch signaling pathway is essential for the EHT; however, its activity needs to be downregulated for HSCs to emerge (Gama-Norton et al., 2015), which is reminiscent of the downregulation of *Gata3*. Indeed, both *Notch1* and one of its important downstream targets, *Hey2*, were upregulated in *Gata3*-GFP^+^ ECs.

### *Gata3* expression correlates with a more quiescent cell state

Among the most prominent gene ontology terms associated with the genes downregulated in *Gata3*-GFP^+^ ECs were proliferation and positive regulation of the cell cycle (Figure S3). We interrogated the differentially expressed genes specifically for cell cycle regulators and uncovered as a general trend an upregulation of cell cycle inhibitors and a downregulation of cell cycle promoters in *Gata3*-GFP^+^ cells (Figure 3E). Furthermore, we observed a significant enrichment of quiescent cells in the *Gata3*-GFP^+^ subsets (Figure 3F). Since the cell cycle inhibitor *Cdkn1c* was upregulated in both *Gata3*-GFP^+^ cell types (Figures 3E and 3G; Table S1), we hypothesized that it was responsible for the quiescent phenotype. Indeed, there was a decrease in *Gata3*-GFP^+^ ECs and HCs in the G0/G1 cell cycle phase from *Cdkn1c*-deficient embryos (Figure 3H). We also noticed that *Gata3*-GFP^+^ ECs were generally more quiescent than HCs, suggesting that HECs may exit the cell cycle to undergo the massive morphological changes required for their transition into HCs, with emerging HCs then starting to proliferate to expand the pre-HSC pool, as reported by others (Batsivari et al., 2017; Oatley et al., 2020; Rybtsov et al., 2016). Interestingly, an upregulation of *RUNX1C*, which is normally upregulated toward the end of EHT, has been associated with an exit from the cell cycle in undifferentiated human pluripotent stem cells (Fidanza et al., 2017). These cells, in which *RUNX1* was activated via dCAS9 promoter targeting (UniSam system) also had higher levels of *GATA3* (Figure 3I). This not only confirms the association of *Gata3* expression with a more quiescent state in different models and species, but may also point to a direct link between these two EHT-associated genes.

### GATA3 function in ECs is required for normal HSPC numbers

**Figure 4:**
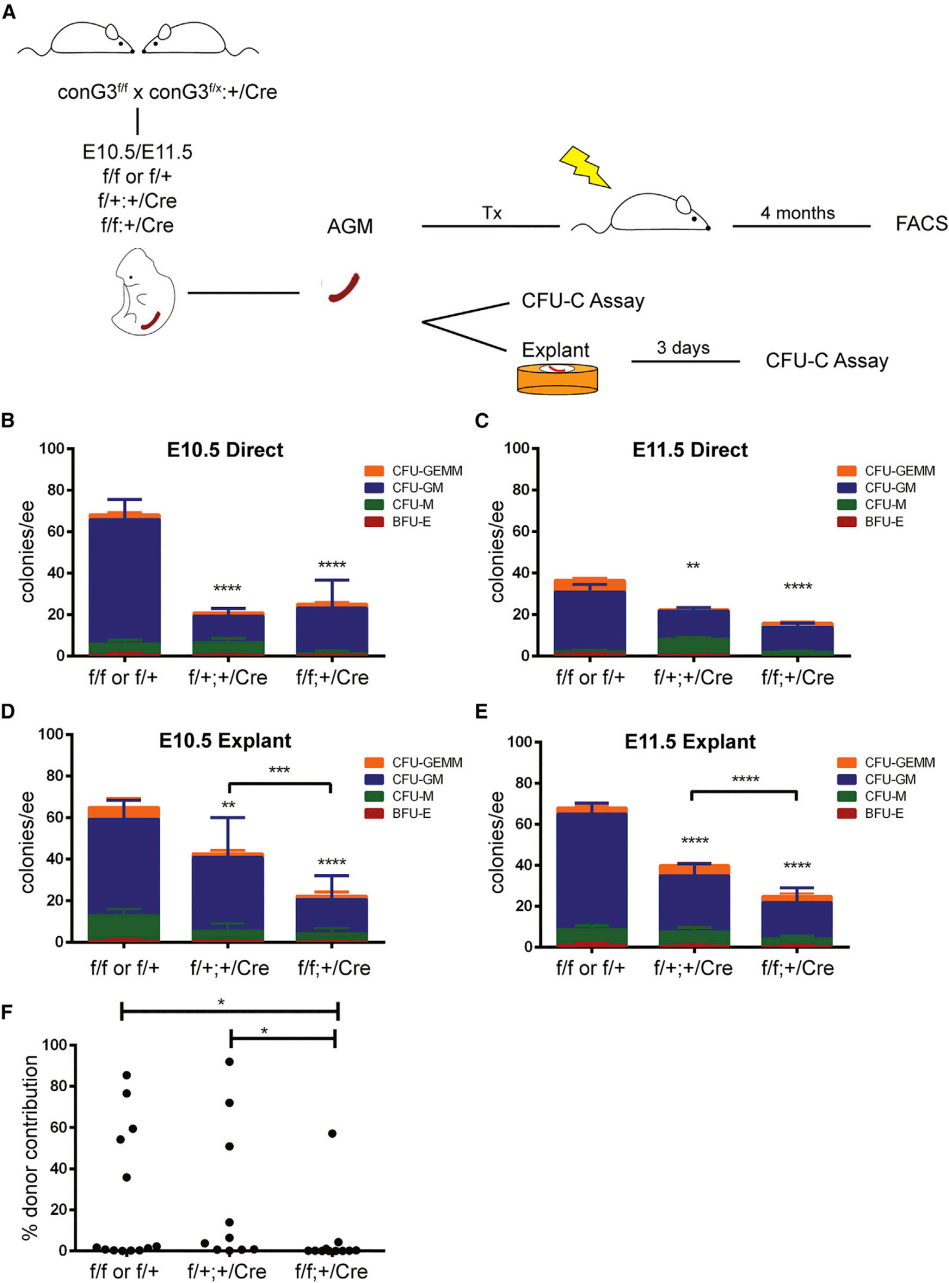
GATA3 function in endothelial cells is required for normal HSPC numbers (A–E) Outline of experiments performed with EC-specific (VEC-Cre) *Gata3* knockout embryos. Colony numbers obtained with E10.5 (B) and E11.5 (C) uncultured AGMs of the indicated genotypes. Colony numbers obtained with E10.5 (D) and E11.5 (E) AGMs of the indicated genotypes after explant culture. n = 3–4, one embryo per replicate. ^∗∗^p < 0.01; ^∗∗∗^p < 0.001; ^∗∗∗∗^p < 0.0001; two-way ANOVA. (F) Percent donor contribution 4 months post-transplant with E11.5 AGM cells of the indicated genotypes. f/f or f/+ n = 13, f/+:+/Cre n = 10, f/f:+/Cre n = 11, one ee per recipient. ^∗^p < 0.05; Mann-Whitney test. See also Figure S4.

To confirm a functional role for GATA3 in the EHT, we crossed a conditional *Gata3* knockout line (Zhu et al., 2004) with a VEC-Cre line (Chen et al., 2009) to delete *Gata3* specifically within the endothelial lineage and its derivatives and assessed how this affected HSPC numbers in the AGM (Figure 4A). Heterozygous and homozygous deletion of *Gata3* significantly reduced progenitor numbers at both E10.5 and E11.5, demonstrating that EC-specific *Gata3* expression is required for HSPC formation and that this is dose dependent (Figures 4B and 4C). A complete knockout of *Gata3* resulted in a very similar reduction of progenitors, showing that it is the expression of *Gata3* in ECs that is important in this context (Figures S4A and S4B). We had previously reported that explant-culturing of AGMs rescued the HSC defect in *Gata3*^*+/−*^ embryos (Fitch et al., 2012). To see if that could also rescue the progenitor defect, we added a 3-day explant culture step. Progenitor numbers in VEC-Cre+ *Gata3*^*+/−*^ AGMs recovered slightly, as there was now a statistically significant difference between heterozygous and homozygous knockout AGMs (Figures 4D and 4E). This was also the case with germline-deleted embryos (Figures S4C and S4D). A substantial number of progenitors remain in AGMs where both copies of *Gata3* were deleted from ECs (Figures 4B–4E); however, genotyping of individual colonies revealed that seven of 20 progenitors had escaped deletion (Figure S4E). This suggests that the effect on progenitors shown here is likely an underestimate, although germline-deleted embryos also retained some GATA3-independent progenitors (Figures S4A–S4D).

AGMs were also transplanted to determine the effect of endothelial-specific *Gata3* deletion on HSC numbers. Repopulation activity was significantly reduced in homozygous knockout AGMs, demonstrating that *Gata3* expression in the aortic endothelium is required for HSC generation (Figure 4F).

## Discussion

This newly described cell-intrinsic role of GATA3 in the EHT adds another facet to the complex way in which GATA3 promotes HSC production in the AGM. We have previously demonstrated that GATA3 is expressed in two AGM niche compartments, the SNS (Fitch et al., 2012) and the sub-aortic mesenchyme (Fitch et al., 2020), from where it also supports emerging HSCs. The relative contribution to AGM hematopoiesis of GATA3 in these three different compartments requires further dissection, although its role in HECs is likely to be dominant as the effect of the EC-specific knockout on HSPC numbers closely mirrors that of the germline knockout (Fitch et al., 2012, and this manuscript). Yet, this defect was rescued through the administration of catecholamine derivatives (Fitch et al., 2012), which could be explained by the fact that HSC production in *Gata3*-null AGMs is not entirely disrupted, with the remaining HSC activity being amplified through catecholamine addition. The function of GATA3 in HECs is also likely to temporally precede its role in supporting HSCs via catecholamine production. As we show here, *Gata3* is already expressed at E9.5 in pro-HSCs, while the maturation of neural crest cells into catecholamine-secreting SNS cells only commences from E10.5. Furthermore, we have previously unveiled a reduction in HECs and intra-aortic cluster formation in the absence of GATA3 (Fitch et al., 2012), suggesting a role for GATA3 at the early stages of EHT initiation.

Another interesting question is the relationship of GATA3 with the other two major regulators of HSC emergence, GATA2 and RUNX1. We showed that GATA3 is required for *Gata2* expression in the SNS, but not in the endothelium, and the expression of *Runx1* in the AGM is reduced by half in *Gata3*-null embryos (Fitch et al., 2012, 2020); however, deletion of RUNX1 and GATA2 from ECs has a much more profound negative impact on HSPC numbers (Chen et al., 2009; de Pater et al., 2013). Data from a recent single-cell RNA-seq (scRNA-seq) analysis of aortic EC subpopulations show an earlier upregulation of *Gata3* in arterial ECs as compared with *Runx1*, *Gata2,* and *Gfi1* (Hou et al., 2020). Instead, it seems to closely mirror the expression pattern of *Hey2*, *Sox7,* and *Sox17*, which need to be downregulated for HSCs to mature (Lizama et al., 2015). This expression pattern of *Gata3* is conserved in humans (Calvanese et al., 2022).

*Gata3* has been shown to be expressed in adult quiescent LT-HSCs where it regulates their entry into the cell cycle (Frelin et al., 2013; Ku et al., 2012). scRNA-seq datasets from mouse embryos and human PSCs revealed that EC and HEC are more quiescent, but enter the cell cycle at the end of the EHT process (Canu et al., 2020; Fadlullah et al., 2022; Oatley et al., 2020; Zeng et al., 2019). The subsequent increase in cycling as HSC precursors mature is also supported by data from a cell cycle reporter mouse (Batsivari et al., 2017) and coincides with the stage at which *Gata3* becomes downregulated. Importantly, when cell cycle progression of PSC-derived ECs was chemically blocked, these cells could no longer generate HSPCs, implying that cell cycle entry is required to complete EHT. Taking all of these data into account, GATA3 may thus keep ECs/HECs quiescent while they undergo the major morphological changes required for the transdifferentiation into HCs, and then, in analogy to its reported role in adult HSCs, may promote their re-entry into the cell cycle for completion of the EHT.

## Experimental procedures

### Mice

All animal work was carried out under a UK Home Office-approved license and following local ethical approval. Males and females of *Gata3-LacZ* knockin mice (van Doorninck et al., 1999), *Gata3-GFP* knockin mice (Grote et al., 2006), conditional *Gata3* knockout mice (Zhu et al., 2004), *p57Kip2* knockout mice (Zhang et al., 1997), VEC-Cre transgenic mice (Chen et al., 2009), and C57BL/6J mice were crossed to obtain embryos of the desired stage and genotype. The day of vaginal plug detection was considered as E0.5. The developmental stage of embryos was specified by either counting somite pairs (E9.5–E10.5) or by eye pigmentation (E11.5). Embryos smaller than their littermates or lacking a heartbeat were excluded.

### AGM explant cultures

AGMs were dissected and cultured on Durapore filters (Millipore) on M5300 long-term culture medium (Stem Cell Technologies) supplemented with 10^−6^ M hydrocortisone (Sigma). After 3 days, AGMs were dissociated with 0.125% collagenase (Alfa Aesar).

### OP9 co-cultures

These were carried out according to Swiers et al. (2013), with further details provided in the supplemental experimental procedures.

### Co-aggregation cultures

AGM cells from E9.5–11.5 embryos were sorted and co-aggregated as one ee with OP9 cells according to Rybtsov et al. (2011). Cultured aggregates were dissociated using collagenase and either analyzed by flow cytometry or transplanted into irradiated mouse recipients.

### Colony-forming assays

Dissociated AGM cells were plated in triplicate (65,000 cells/plate) in methylcellulose (M3434; Stem Cell Technologies), incubated at 37°C, and colonies scored 7 days later.

To detect *Gata3* deletion by VEC-Cre, individual colonies were picked and analyzed by PCR, using the following primers: forward, CAGTCTCTGGTATTGATCTGCTTCTT; and reverse, GTGCAGCAGAGCAGGAAACTCTCAC.

### Transplantations

Single-cell suspensions were injected intravenously into irradiated (split dose of 460–475 rad, Cesium source) recipients together with 2 × 10^5^ spleen cells (for direct transplantations) or 2 × 10^4^ bone marrow cells (for co-aggregates). Recipients were CD45.1/.2 or CD45.1/.1 and the donors were CD45.2/.2 on a C57BL6J background. Recipient blood was analyzed at 1 and 4 months post-transplantation.

### Flow cytometry

Antibody stainings were performed 30 min on ice in the dark, with further details on antibodies, controls, and equipment provided in the supplemental experimental procedures.

For cell cycle analysis, sorted cells were incubated for 1 min at room temperature 1:1 with DAPI staining solution (5 μg/mL DAPI [Sigma] and 1% [v/v] Nonidet P40 [Sigma] in dH2O) and analyzed on an LSRFortessa (BD Bioscience).

### Immunohistochemistry

Cryosections were prepared and stained as described previously (Fitch et al., 2012), with further details provided in the supplemental experimental procedures.

### X-gal staining

*Gata3*^*lz/+*^ embryos were fixed for 1 h with PBS/10% Formal Saline/0.2% Glutaraldehyde/2 mM MgCl2/5 mM EGTA/0.02% NP40 at 4°C and stained overnight at room temperature with 5 mM K3Fe(CN)6/5 mM K4Fe(CN)6/2 mM MgCl2/0.01% Nadeoxycholate/0.02% NP40/0.1% X-gal (all in PBS). Stained embryos were cryopreserved and sectioned as above and sections counterstained with Neutral Red.

### Endogenous gene activation in human iPSCs

Human iPSCs were cultured and transfected with the *RUNX1C*-activating UniSAM system as described previously (Fidanza et al., 2017), with experimental details and qPCR primers provided in the supplemental experimental procedures.

### RNA sequencing

Libraries for RNA-seq were prepared according to the protocol by Picelli et al. (2014). Cells were initially sorted from three to five biological replicates into tubes based on their populations: *Gata3*-GFP^+^ EC, *Gata3*-GFP^−^ EC, *Gata3*-GFP^+^ HC and *Gata3*-GFP^−^ HC. Each population was then sorted again into a 96-well plate, 20 cells/well (20 pools per population), containing 2.3 μL of lysis buffer (0.2% RNase inhibitor [Ambion, Thermo Fisher Scientific] in Triton X-100 [Sigma]). Details on the reverse transcription, PCR pre-amplification, library preparation steps and data analysis are provided in the supplemental experimental procedures.

### Statistical analysis

Graph preparations and statistical analysis were performed using GraphPad Prism. The Mann-Whitney test was used for transplantation experiments, paired t test for colony-forming assay following OP9 co-culture and co-aggregates, and two-way ANOVA test for colony-forming assays.

### Data and code availability

The data have been deposited in NCBI’s Gene Expression Omnibus (GEO) repository under accession number GSE114926.

## Author contributions

N.Z. performed and designed most experiments; E.D. and R.H. performed bioinformatics analyses; N.K.W. and B.G. provided advice and assistance with the scRNA-seq experiment; L.N. and A.F. performed experiments; L.M.F. provided important reagents; K.O. conceived and supervised the study and wrote the manuscript.

## Supplemental information

Document S1. Figures S1–S4 and supplemental experimental procedures

Table S1.Differentially expressed genes in Gata3-GFP+/− hematopoietic (HC) and endothelial (EC) cells Supplied as a separate Excel spreadsheet

Document S2. Article plus supplemental information
