# Supplementary material for: Endothelial-specific Gata3 expression is required for hematopoietic stem cell generation
Source: Stem Cell Reports. 2022 Jul 28;17(8):1788–98. doi: 10.1016/j.stemcr.2022.06.008 (PMC9391417; doi:10.1016/j.stemcr.2022.06.008)
Supplement: Document S1. Figures S1–S4 and supplemental experimental procedures [file mmc1.pdf]

**Stem Cell Reports, Volume *17***

## Supplemental Information

# Endothelial-specific Gata3 expression is required for hematopoietic stem cell generation

**Nada Zaidan, Leslie Nitsche, Evangelia Diamanti, Rebecca Hannah, Antonella Fidanza, Nicola K. Wilson, Lesley M. Forrester, Berthold Göttgens, and Katrin Ottersbach**

**Supplemental Figures**

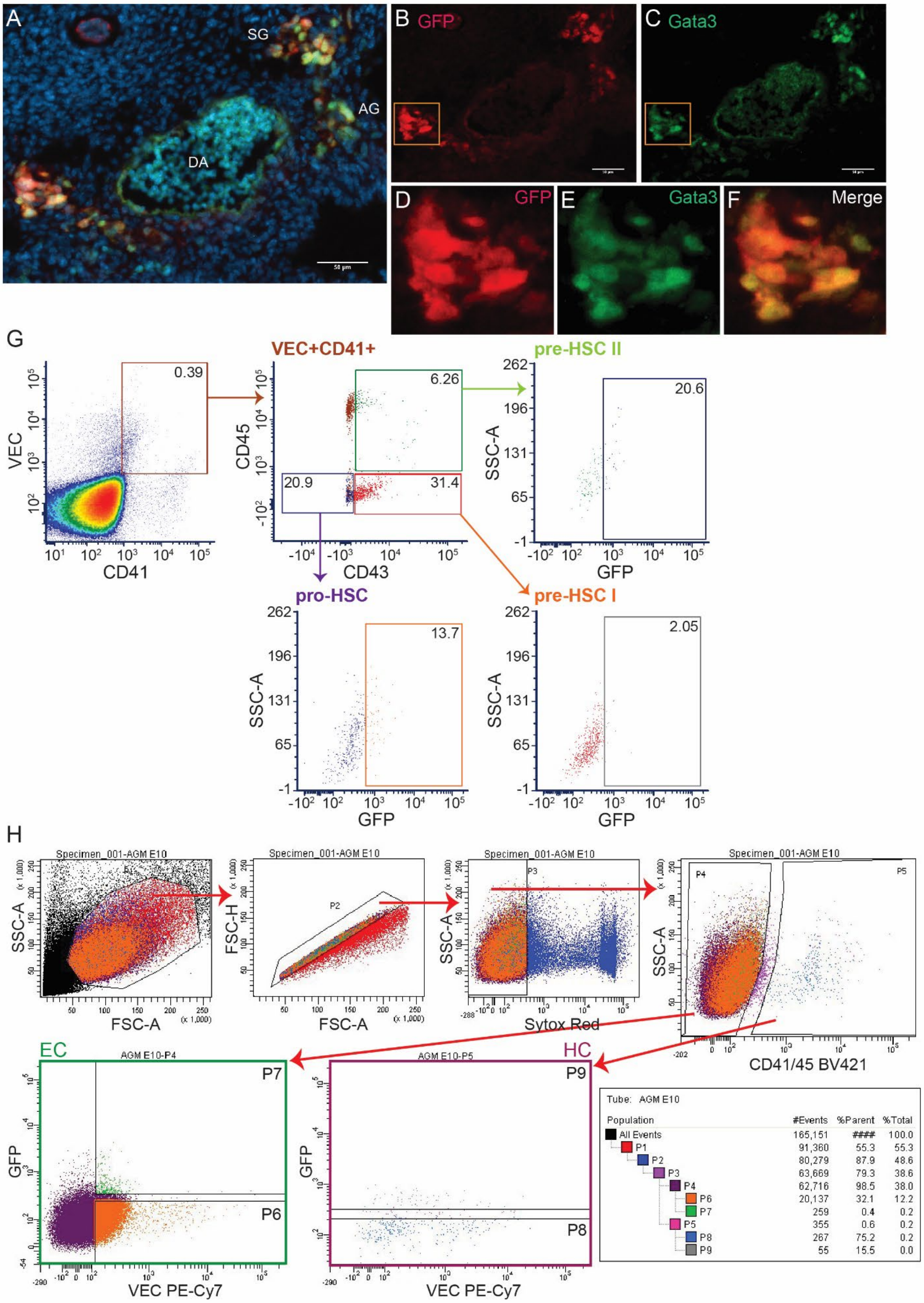


Average cell number per embryo

| EC | | HC | |
|---|---|---|---|
| Gata3-GFP+ | 60.8 | Gata3-GFP+ | 38.7 |
| Gata3-GFP- | 1507.7 | Gata3-GFP- | 1168.7 |

**Figure S1 *Gata3*-GFP expression mirrors that of endogenous GATA3 protein and is expressed in HSC precursors – related to Figure 1 and 2**

Cryosection from a *Gata3*-GFP+ E11.5 embryo showing the co-staining between GATA3 (green) and GFP (red) with DAPI as nuclear stain in blue. (**A**) merged image, (**B**) antibody to GFP, (**C**) antibody to GATA3. (**D-F**) Magnification of area indicated by yellow box in B and C demonstrating co-staining between GATA3 and GFP. DA: dorsal aorta, AG: adrenal anlage, SG: sympathetic ganglia. (**G**) Flow cytometry gating strategy for detecting *Gata3*-GFP expression in pro-HSCs (VEC+CD41+CD43-CD45-), pre-HSC I (VEC+CD41+CD43+CD45-) and pre-HSC II (VEC+CD41+CD43+CD45+). (**H**) Gating strategy for flow cytometry sorting of *Gata3*-GFP+/- ECs (VEC+ CD41/45-) and HCs (CD41/45+) for the CFU-C assays (Fig.1E) and the co-culture experiments (Fig. 2A). Average number of cells per embryo are stated underneath.

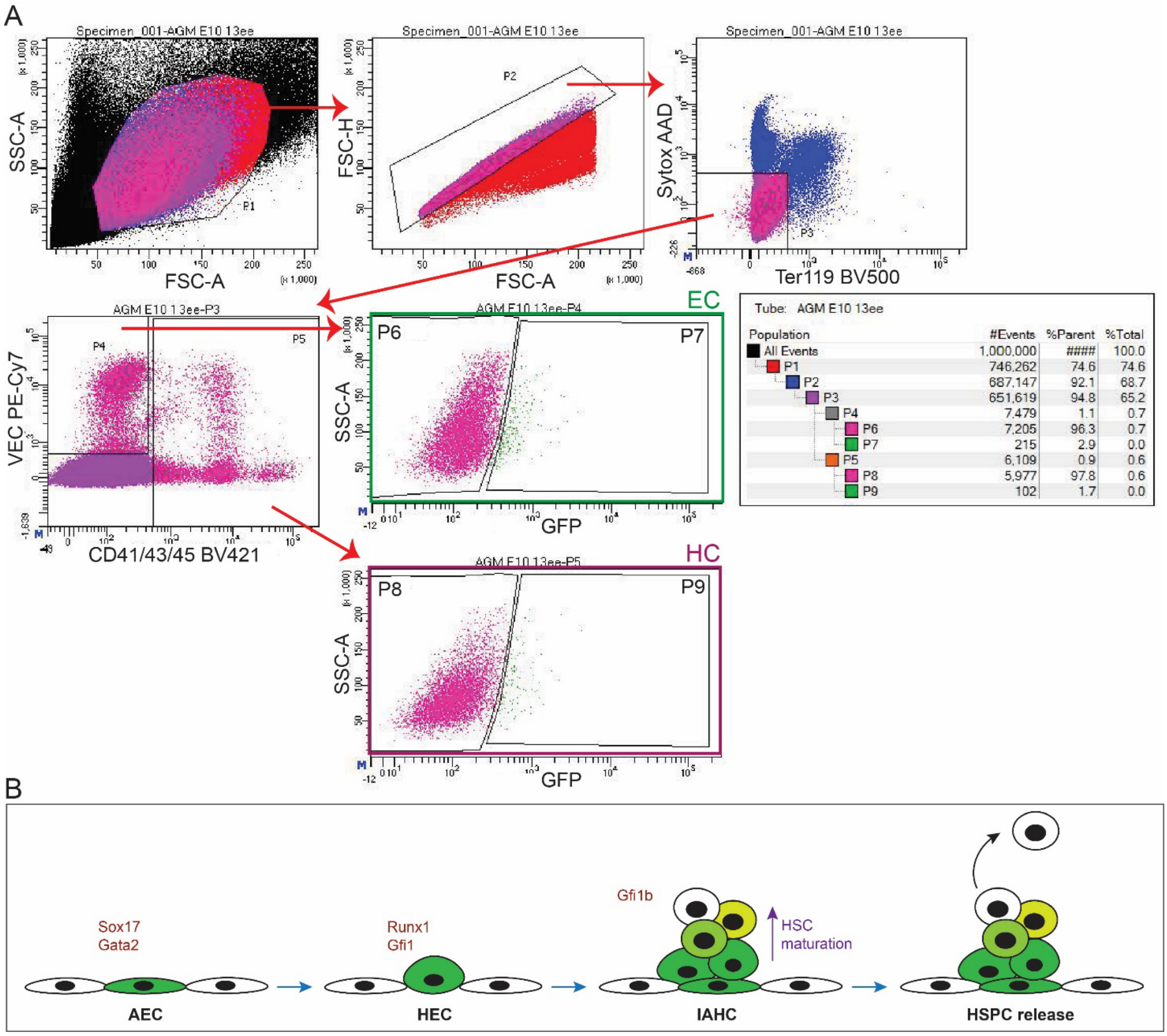


**Figure S2 *Gata3*-GFP+/- EC and HC sorting strategy – related to Figure 2 and 3**

(**A**) Gating strategy for flow cytometry sorting of *Gata3*-GFP+/- ECs (VEC+ CD41/43/45- Ter119-) and HCs (CD41/43/45+ Ter119-) for the co-aggregate experiments in Figure 2J. (**B**) Schematic diagram of *Gata3* expression (green) during the endothelial-to-hematopoietic transition, with upregulation of other key transcription factors indicated in red. *Gata3* is expressed in arterial endothelial cells (AEC), in which a hematopoietic transcriptional program is then switched on as they become hemogenic endothelial cells (HEC). These then mature into hematopoietic stem and progenitor cells (HSPC) inside intra-aortic hematopoietic clusters (IAHC) via pro-HSC, pre-HSC I and pre-HSC II stages, during which *Gata3* is downregulated until it is no longer expressed in HSPCs released into the circulation.

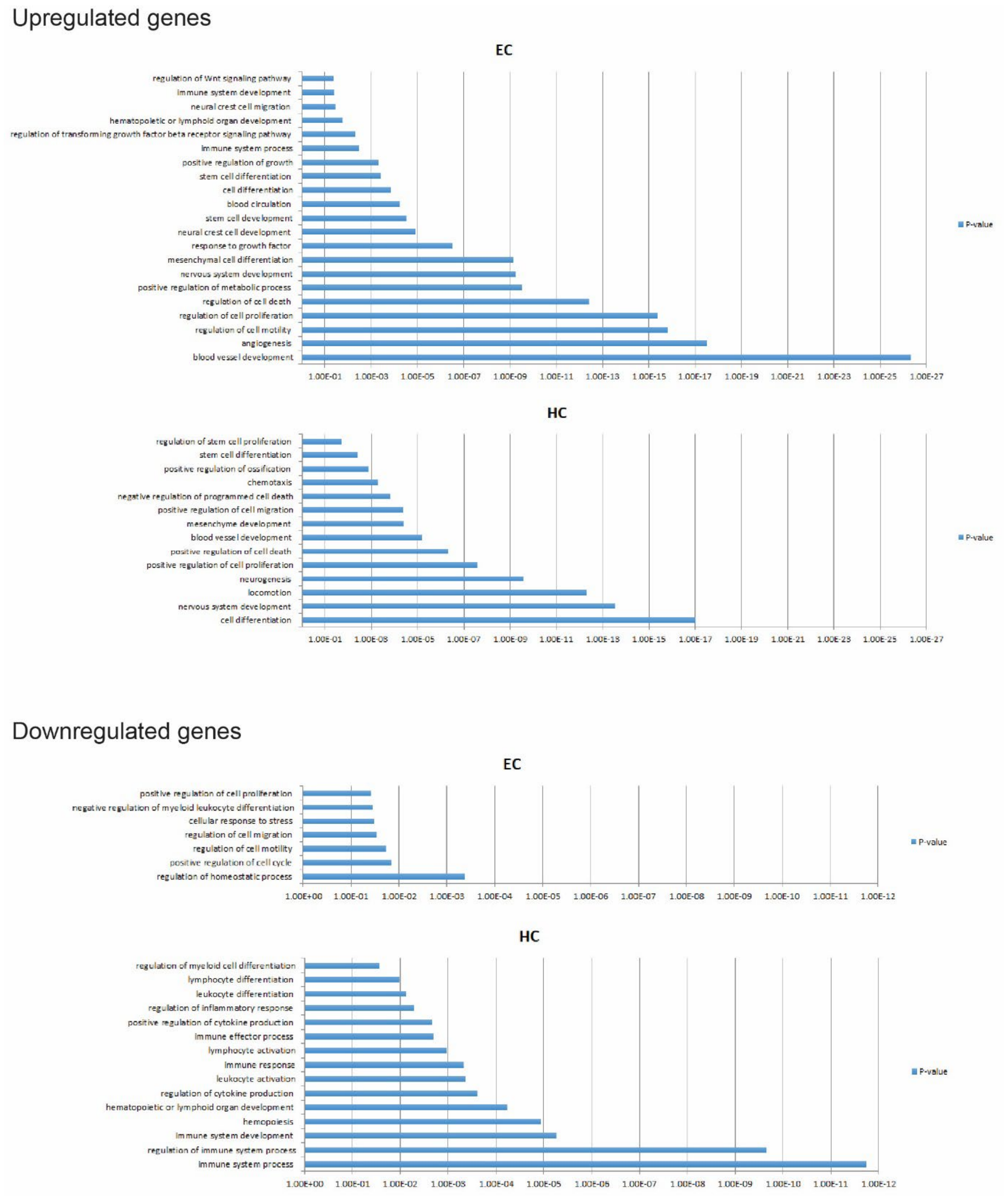


**Figure S3 Gene ontology terms enriched in differentially expressed genes – related to Figure 3**

GO terms significantly enriched amongst the genes upregulated and downregulated in *Gata3*-GFP+ ECs and HCs.

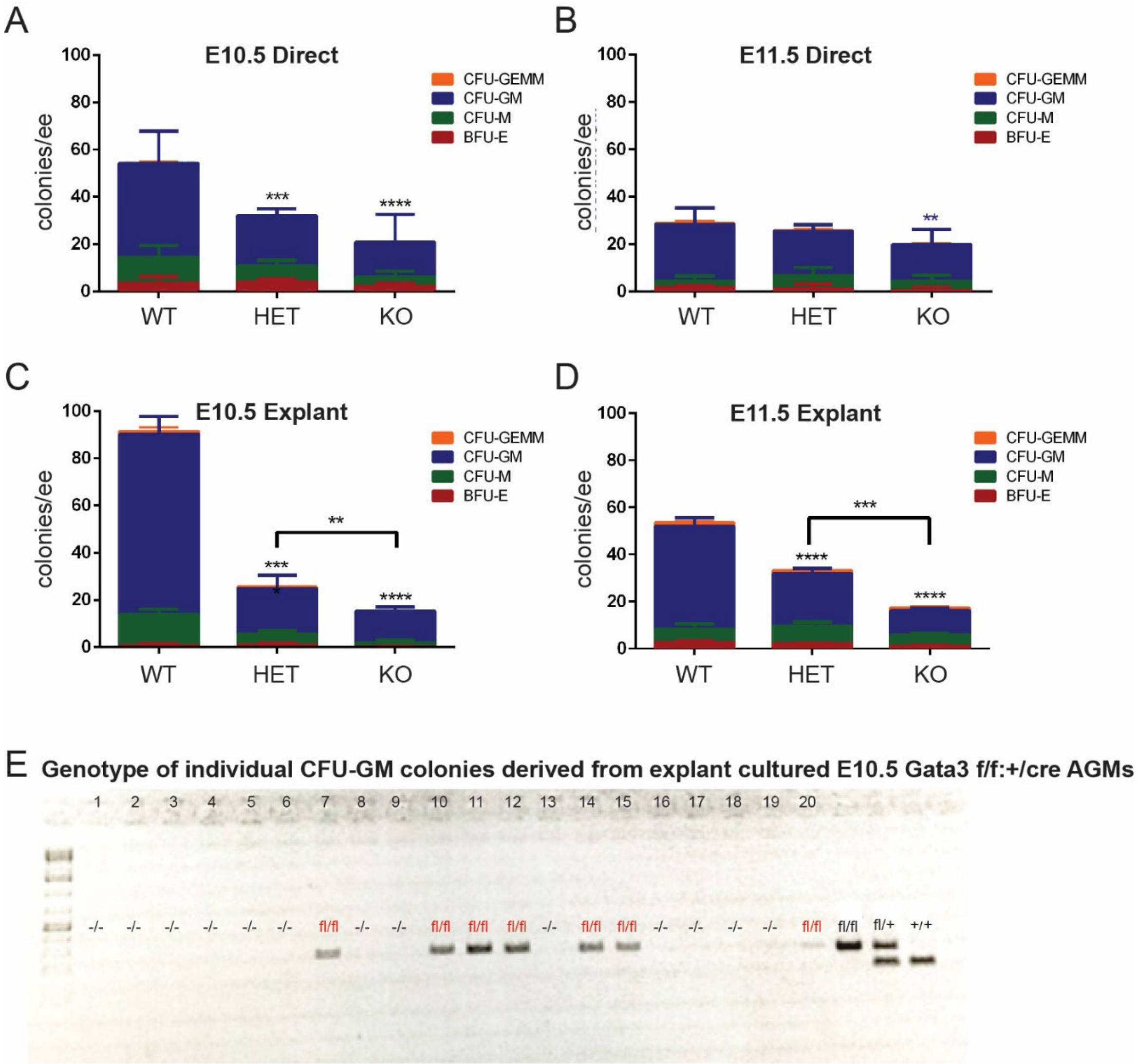


**Figure S4 Colony-forming assays with *Gata3* germline-deleted AGMs – related to Figure 4**

CFU-C assays of *Gata3*$^{+/+}$ (WT), *Gata3*$^{+/-}$ (HET) and *Gata3*$^{-/-}$ (KO) uncultured E10.5 (**A**) and E11.5 (**B**) AGM cells and following 3 days of explant culture of E10.5 (**C**) and E11.5 (**D**) AGMs. Results are from 3-4 independent experiments (n=3-4, 1 embryo per replicate). ** p<0.01; *** p<0.001; **** p<0.0001; Two-way ANOVA. (**E**) Gel electrophoresis result from the genotyping of 20 individual CFU-GM colonies picked from methylcellulose plates seeded with cells from *Gata3-fl/fl; VEC-Cre+* AGMs cultured as explants, demonstrating that some cells retained the floxed allele (highlighted in red) and therefore had escaped recombination and deletion of *Gata3*. n=20.

**Supplemental Table S1**

Differentially expressed genes in *Gata3*-GFP+/- hematopoietic (HC) and endothelial (EC) cells. Supplied as a separate Excel spreadsheet.

**Supplemental Experimental Procedures**

**OP9 co-cultures**

These were carried out according to Swiers et al., 2013, with further details provided in the Supplemental Experimental Procedures. OP9 cells were maintained in $\alpha$MEM (Gibco) with 20% heat deactivated FCS (Hyclone) and 0.22% sodium bicarbonate (Gibco) at 37°C with 5% CO2. 24h prior to the start of the co-cultures, cells were plated in $\alpha$MEM with 10% FCS and 0.01% of 2-mercaptoethanol. Sorted hematopoietic and endothelial cell populations were plated on confluent OP9 stroma. Cultures were supplemented with SCF, FLTe3 ligand and IL7 at 10ng/ml (all from Pepro-Tech) and incubated for 8–10 days at 37°C, 5% $CO_2$. Hematopoietic output was assessed by CFU-C and flow cytometry.

**Flow cytometry**

Antibody stainings were performed 30min on ice in the dark, using the following antibodies: CD41-BV421 (1:100; Biolegend; cat# 133911), CD45-BV421 (1:100; Biolegend; cat# 103133), CD45-APC-Cy7 (1:100; BD Bioscience; cat# 561037), CD45-A700 (1:50; Biolegend; cat# 103128), CD45.1-PE (1:200; eBioscience; cat# 12-0453-82), CD45.2-A700 (1:200; Biolegend; cat# 109821), CD43-BV421 (1:100; BD Bioscience; cat# 752957), Ter119-V500 (1:100; BD Bioscience; cat# 562120), VEC-PE-Cy7 (1:100; Biolegend; cat# 138016), VEC-AF647 (1:100; BD Bioscience; cat# 562242), CD11b-PB (1:200; Biolegend; cat# 101224) and Gr1-PB (1:200; Biolegend; cat# 108429). Peripheral blood samples from transplant recipients were pre-treated with Red Cell Lysis buffer (BD Bioscience). Dead cells were excluded via 7-aminoactinomycin D staining 1:1000 (7AAD, Invitrogen) or Sytox AAD (1:5000) (Invitrogen). All experiments included the following controls: unstained cells, single stained samples and fluorescent minus one (FMO) controls. Cells were analyzed using LSRFortessa (BD Bioscience) or sorted using MoFlo (Beckman Coulter), ARIA (BD Bioscience), or Fusion (BD Bioscience) and data analyzed with the FlowJo software (BD Bioscience).

**Immunohistochemistry**

Embryos were fixed in 2% paraformaldehyde (Sigma) in PBS for 1.5h at 4°C, cryoprotected overnight in 30% sucrose/PBS at 4°C and embedded in OCT TissueTek. 10$\mu$m sections were prepared on a cryostat (Leica, CM3050 S).

For antibody staining, either an Avidin/biotin system or fluorescent-labelled secondary antibodies were used. Cryosections were blocked with 200$\mu$l of PBS/0.05% Tween/1% BSA, containing Avidin/biotin block where appropriate, and incubated with primary antibody for 24 hours at 4°C in the dark. The next day, the slides were incubated with secondary antibody or fluorescently labelled streptavidin for 45min at room temperature in the dark and then mounted with Vectashield containing DAPI (Vectorlabs). The following

antibodies were used: TH (mouse; 1:300; Millipore; cat# MAB318), GFP (chicken; 1:400; Thermofisher; cat# 600-901-215), GFP (rabbit; 1:500; Life Technologies; cat# A11122), CD34-FITC (rat; BD Bioscience; 1:100; cat# 560238), Gata3 (goat; 1:300; BD Bioscience; cat# AF2605), anti-chicken-Alexa647 (1:500; Jackson Immunoresearch; cat# 703-605-155), anti-mouse-Alexa546 (1:200; Life Technologies; cat# A10036), anti-rabbit-Alexa647 (1:200; Life Technologies, cat# A31573), anti-rabbit-Alexa555 (1:500; Life Technologies; cat# A31572), and anti-goat CF 633 (1:200; Sigma; cat# SAB4600128). Images were acquired on a Leica SP8 confocal microscope and analyzed with Leica Las X software.

**Endogenous gene activation in human iPSCs**

Human iPSCs were cultured in StemPro hESC SFM (Gibco) supplemented with 20ng/ml bFGF (R&D) on CELLstart (Gibco) coated wells. Single cell suspension was obtained using Accutase (Gibco) and $3 \times 10^5$ cells were reverse transfected with 2µg of UniSAM DNA using the Xfect Transfection reagent (Clontech) and plated into a coated 6 well plate. Each well was transfected with the PB-UniSAM plasmid (Addgene 99866 (Fidanza et al., 2017) containing either one of the four gRNAs against *RUNX1C* promoter (Fidanza et al., 2017)), which results in activation and upregulation of *RUNX1C*, or no guide (Empty vector control). Total RNA was extracted using the RNAeasy Mini Kit (Qiagen) 2 days post transfection and cDNA synthesized using the High-Capacity cDNA synthesis Kit (Applied Biosystem). Gene expression analysis was performed in triplicate using the LightCycler 384 (Roche) with SYBR Green Master Mix II (Roche), β-Actin was used as reference genes. Gene activation values were calculated as fold change relative to the empty vector control group. The following primers were used: RUNX1C_fw agcctggcagtgtcagaagt, RUNX1C_rv gggactcaatgatttcttttacca, GATA3_fw gctcttcgctacccaggtg, GATA3_rv gtaaaaaggggcgacgactc, ACTB_fw ccaaccgcgagaagatga, ACTB_rv ccagaggcgtacagggatag.

**RNA Sequencing**

*Reverse transcription*

For the reverse transcription step, 2µl of annealing mix (5% ERCC RNA spike-In Mix (pre-diluted at 1:25,000; Invitrogen), 5% Oligo-dT (5'–AAGCAGTGGTATCAACGCAGAGTACT30VN-3'; 100µM; biomers.net), 50% dNTP 10mM (Fermentas) and 40% distilled water) were added to each well and the plates incubated at 72°C for 3min and immediately placed on ice. 5.7µl of reverse transcription mixture (0.5µl Superscript II RT (200 U/µl; Invitrogen), 0.25µl RNase inhibitor (20 U/µl), 2µl 5x Superscript II First Strand Buffer (Invitrogen), 0.5µl DTT (Invitrogen), 2µl 100µM Betaine (Sigma), 0.06µl 1M MgCl2 (Ambion), 0.1µl TSO Oligo (5'-AAGCAGTGGTATCAACGCAGAGTACATrGrG+G-3'; 100µM; Exiqon) and 0.29µl distilled water) were added and the plate placed in a PCR cycler: 42°C for 90min, 10 cycles of: 50°C for 2min, 42°C for 2min, then at the end 70°C for 15min.

*PCR Pre-amplification*

For the PCR Amplification, 15µl of the PCR mixture were added per well, consisting of 12.5µl KAPA HiFi Hotstart ReadyMix (2x; KAPA Biosystems), 0.25µl ISPCR primer (5′-AAGCAGTGGTATCAACGCAGAGT-3′; 10µM; biomers.net) and 2.25µl distilled water. The reactions were run at 98°C for 3min, 21 cycles of: 98°C for 20sec, 67°C for 15sec, 72°C for 6min, and 72°C for 5min at the end.

Ampure XP beads (Beckman Coulter) were used for PCR clean up. The size distribution of the cDNA library was checked on an Agilent high-sensitivity DNA chip (Agilent Technologies), according to the manufacturer instructions.

*Sequencing library preparation*

Tagmentation was carried out using the Illumina Nextera XT DNA sample preparation kit (Illumina) according to an optimized Tagmentation protocol (Fluidigm). Index Primers 1 (N701-N712) and 2 (S501-S508) at a ratio of 12.5% (each) were combined that each well was uniquely labelled and dual-indexing metadata could be obtained (Nextera XT 96-Index kit; Illumina). Following PCR amplification, libraries were pooled and cleaned up with Ampure XP beads. The library size distribution was checked on an Agilent high-sensitivity DNA chip and the library quantified using the KAPA library quantification kit (KAPA Biosystems). Pooled libraries were sequenced on an Illumina Hi-Seq 4000 (Sanger, Cambridge), as single-end 125 base pair reads.

*Sequencing data analysis*

The data were aligned using STAR (Dobin et al., 2013) to Ensembl genome build 81 (Zerbino et al., 2018), with gene counts obtained using HT-Seq (Anders et al., 2015). Quality control filtering and normalization was performed in R. More than 500,000 reads uniquely mapped (either to ERCC spike-ins or endogenous mRNA), with more than 20% of total reads mapped to mRNA, less than 20% of mapped reads allocated to mitochondrial genes, less than 20% of reads mapped to ERCC spike-ins and more than 8000 high coverage genes. Cells were normalized with Scran (Lun et al., 2016) and highly variable genes identified estimating technical variance with the ERCC spike-ins (Brennecke et al., 2013). In-house programs in R were used for PCA and t-distributed Stochastic Neighbor Embedding (t-SNE) dimensionality reduction. Genes differentially expressed between cell types and Gata3 expression groups were identified using the rank_genes_groups function with the t-test_overestim_var method. P-values were adjusted using the benjamini-hochberg procedure, and genes with adjusted p-value < 0.01 considered as significant.

Gene Ontology (GO) analysis was performed using the Gene Ontology Consortium Enrichment Analysis, which utilizes PANTHER Classification System for biological processes in mus musculus.

**Supplemental References**


Anders, S., Pyl, P.T., and Huber, W. (2015). HTSeq--a Python framework to work with high-throughput sequencing data. Bioinformatics *31*, 166-169.

Brennecke, P., Anders, S., Kim, J.K., Kolodziejczyk, A.A., Zhang, X., Proserpio, V., Baying, B., Benes, V., Teichmann, S.A., Marioni, J.C.*, et al.* (2013). Accounting for technical noise in single-cell RNA-seq experiments. Nat Methods *10*, 1093-1095.

Dobin, A., Davis, C.A., Schlesinger, F., Drenkow, J., Zaleski, C., Jha, S., Batut, P., Chaisson, M., and Gingeras, T.R. (2013). STAR: ultrafast universal RNA-seq aligner. Bioinformatics *29*, 15-21.

Fidanza, A., Lopez-Yrigoyen, M., Romano, N., Jones, R., Taylor, A.H., and Forrester, L.M. (2017). An all-in-one UniSam vector system for efficient gene activation. Sci Rep *7*, 6394.

Lun, A.T., Bach, K., and Marioni, J.C. (2016). Pooling across cells to normalize single-cell RNA sequencing data with many zero counts. Genome Biol *17*, 75.

Picelli, S., Faridani, O.R., Bjorklund, A.K., Winberg, G., Sagasser, S., and Sandberg, R. (2014). Full-length RNA-seq from single cells using Smart-seq2. Nat Protoc *9*, 171-181.

Swiers, G., Baumann, C., O'Rourke, J., Giannoulatou, E., Taylor, S., Joshi, A., Moignard, V., Pina, C., Bee, T., Kokkaliaris, K.D.*, et al.* (2013). Early dynamic fate changes in haemogenic endothelium characterized at the single-cell level. Nat Commun *4*, 2924.

Zerbino, D.R., Achuthan, P., Akanni, W., Amode, M.R., Barrell, D., Bhai, J., Billis, K., Cummins, C., Gall, A., Giron, C.G.*, et al.* (2018). Ensembl 2018. Nucleic Acids Res *46*, D754-D761.
